# Stereotactic body radiotherapy for metastatic sarcoma to the lung: adding to the arsenal of local therapy

**DOI:** 10.1186/s13014-023-02226-3

**Published:** 2023-03-01

**Authors:** Paulina M. Gutkin, Elizabeth Gore, John Charlson, John C. Neilson, Candice Johnstone, David M. King, Donald A. Hackbarth, Adam Wooldridge, Rajeev Mannem, Meena Bedi

**Affiliations:** 1 Department of Radiation Oncology, Medical College of Wisconsin, 8800 West Doyne Ave, Milwaukee, WI 53226 USA; 2 Medical Oncology, Medical College of Wisconsin, Wauwatosa, WI USA; 3 Orthopaedic Surgery, Medical College of Wisconsin, Wauwatosa, WI USA; 4 Radiology, Medical College of Wisconsin, Wauwatosa, WI USA

**Keywords:** Metastatic sarcoma, Radiation therapy, Sarcoma, Stereotactic body radiation therapy, SBRT

## Abstract

**Introduction:**

Conventional treatment of pulmonary metastatic sarcoma primarily involves surgery, with systemic therapy added in select patients. However, broader applications of radiation therapy techniques have prompted investigation into the use of stereotactic body radiotherapy (SBRT) for treatment of metastatic sarcoma, an attractive non-invasive intervention with potential for lower rates of adverse events than surgery. Current data are limited to retrospective analyses. This study analyzed 2-year local control and overall survival and adverse events in patients prospectively treated with SBRT to pulmonary sarcoma metastases.

**Methods:**

Patients prospectively treated with SBRT to the lung for biopsy-proven metastatic sarcoma at a single institution from 2010 to 2022 were included. SBRT dose/fractionation treatment regimens ranged from 34 to 54 Gy in 1–10 fractions using photons. Local recurrence, local progression-free survival (LPFS) and overall survival (OS) were calculated from the end of SBRT. Univariable analysis (UVA) was performed using the log-rank test. Multivariable analysis (MVA) was performed using the Cox proportional hazards model. Adverse events due to SBRT were graded based on the Common Terminology Criteria for Adverse Events, version 4.0.

**Results:**

Eighteen patients with metastatic sarcoma were treated to 26 pulmonary metastases. The median local progression-free survival was not met. The median overall survival was not met. The local control rate at 2 years was 96%. 2-year LPFS was 95.5% and OS was 74%. Three patients (16.7%) developed grade 1 adverse events from SBRT. There were no adverse events attributed to radiation that were grade 2 or higher.

**Conclusion:**

We report prospective data demonstrating that SBRT for sarcoma pulmonary metastases affords a high rate of local control and low toxicity, consistent with prior sarcoma SBRT retrospective data. This study adds to the wealth of information on SBRT in a radioresistant tumor. Though largely limited to retrospective reviews, current data indicate high rates of local control with favorable toxicity profiles. Therefore, SBRT for pulmonary sarcoma metastases may be considered for properly selected patients.

**Supplementary Information:**

The online version contains supplementary material available at 10.1186/s13014-023-02226-3.

## Introduction

Sarcomas derive from mesenchymal tissue and represent about 1% of adult tumors. Stage of the tumor largely dictates the likelihood of survival, with metastatic sarcoma carrying a poor prognosis. About 19–50% of patients with localized disease develop metastasis [1, 2], while about 15% present with metastasis at diagnosis [3]. Treatment outcomes for metastatic sarcoma greatly rely on the efficacy of current systemic therapies and treatment intent is often palliative. In appropriately selected patients, local treatment of metastatic disease carries survival benefits [2].

Sarcoma most commonly metastasizes to the lung. Surgical resection, systemic therapy, radiation therapy, and interventional radiology ablative techniques are often used in the management of patients with pulmonary metastasis. However, multiple factors impact intervention for lung metastases, including the number, location, and size of metastatic lesions, as well as patient symptoms and performance status, risk of complications, and patient preference. In addition, if systemic therapy is delivered, it must be interrupted or discontinued for several weeks prior to any surgery to maximize wound healing. Thus, potential for progression during delay of systemic treatment for surgery must be carefully evaluated.

Stereotactic body radiotherapy (SBRT) is a non-invasive radiation therapy technique that delivers highly conformal, high dose radiation, often in only 1 to 5 treatment sessions. Traditionally reserved for patients unfit for surgery, SBRT applications are broadening. Data supporting SBRT as an effective modality for treating primary lung cancers [4] and lung metastases [5] are rapidly expanding; retrospective studies investigating the efficacy of SBRT for non-sarcoma lung metastases have reported local control rates as high as 94%, with minimal adverse side effects [6].

Sarcomas are considered radioresistant, thus radiotherapy is not typically first-line intervention for metastatic disease unless surgical and systemic treatment options fail. Due to advancements in precise delivery of maximal tolerable biologic equivalent doses, the paradigm for the role of radiation in treating historically radioresistant tumors is evolving. SBRT has demonstrated promising local treatment for primary sarcoma histologies [7–9]. In addition, surgery is not always feasible or preferred by patients. In some scenarios, a combined approach with surgery and SBRT may be executed and as such, it is imperative to have robust data to support SBRT as an excellent alternative or compliment to surgery in the sarcoma setting. Herein, we report local progression-free survival and safety using SBRT for STS lung metastases.

## Methods

This study of prospective data was approved by the study site Institutional Review Board. Eight patients were enrolled on a single institution phase II trial between September 2012 and December 2015. The initial phase II trial was closed due to slow accrual, but data from 10 additional patients meeting the same inclusion criteria were prospectively collected from January 2016 through December 2018 and included in this study; these additional patients underwent the same treatment schema ad received the same follow up and assessments as the patients enrolled on the trial. Inclusion criteria consisted of subjects that were > 18 years of age with pathologic confirmation of a primary soft tissue sarcoma of the extremity or chest-wall. Biopsy of each lung metastasis targeted with SBRT was obtained within 16 weeks prior to treatment to confirm sarcoma pathology. Participants were required to have a Karnofsky Performance Status of ≥ 60 and determined to be medically operable. Systemic therapy, if given, must have been completed ≥ 21 days prior to the start of radiotherapy. Patients underwent a 2-week chemotherapy washout period, and no systemic therapy was utilized during SBRT.

Patients with primary sarcoma who developed primary lung cancer and patients with inconclusive lung biopsy results were excluded. Patient demographics, smoking history, primary tumor histology, initial tumor staging, and treatment information were recorded. Tumor metastasis size was collected from the chest CT radiograph before the start of SBRT. Location of lesion was categorized by lung lobe and by location relative to central organs. Lesions were categorized as central if they were within or adjacent to a 2 cm expansion around the proximal bronchial tree or abutted the heart and great vessels. Ultracentral lesions were within 1 cm of the proximal bronchial tree and abutted or invaded the mediastinum, trachea, bronchus or esophagus. Lung lesions were categorized as peripheral if they were > 2 cm from the proximal bronchial tree. Patients were defined as having oligometastatic disease at the time of SBRT if they had ≤ 5 metastatic lesions but this was not a requirement of eligibility.

For intermediate to high grade lesions, surveillance imagining for sarcomas included CT of the chest, abdomen, and pelvis every 4 months for 2 years. Subsequent imaging was completed every 6 months for 3 additional years. For low grade lesions, CT imaging or chest X-ray was obtained every 6 months for 5 years. All patients were discussed at a multidisciplinary tumor board, which included surgical oncologists, medical and radiation oncologists, pathologists, and radiologists. Treatment recommendations were presented to each patient.

### Radiation treatment

Patients were simulated using CT imaging with both 3D and 4D scans to account for respiratory motion. Photon (x-ray) beams with photon energies of 6 MV were used. All patients received a simulation CT scan used to contour the gross tumor volume (GTV). No margin was added for clinical target volume (CTV). GTV were defined either on MIP (Maximum Intensity Projection) images or using GTVs at 3 phases (inspiration, expiration and midphase) of the respiratory cycle to create an ITV. An additional 0.5 cm in the axial plane and 0.5 to 1.0 cm in the longitudinal plane (craniocaudal) was added to the each GTV to create the planning target volume (PTV).

SBRT dose/fractionation treatment regimens ranged from 34 to 54 Gy in 1 to 10 fractions using photons. The treatment goals were 54 Gy in 3 fractions for peripheral lesions and 50 Gy in 5 fractions central lesions. Treatment dose and fractionation was per physician discretion and was adjusted based on tumor size, location, organs at risk, patient comorbidities, and prior treatment. Per protocol, treatments delivered with > 10 Gy per fraction had a minimum of 48-h interfraction interval. Treatments with ≤ 10 Gy per fraction had a minimum 24-h interfraction interval. Treatments were completed over 14 days for 3 fraction treatments and over 21 days for > 5 fraction treatment schedules.

Local recurrence (LR) was assessed radiographically and was defined as tumor recurrence or increased size of the treated tumor, based on Response Evaluation Criteria in Solid Tumors (RECIST) criteria on CT imaging, within the radiation PTV [10]. Time to LR was measured from the end of SBRT to the time of tumor recurrence or last follow up. Local progression-free survival was defined as the time from end of SBRT to the time of LR. Overall survival (OS) was measured from the end of radiation to the time of death.

Adverse radiation events due to SBRT were graded based on the Common Terminology Criteria for Adverse Events, version 4.0.

### Statistical analysis

Prognostic factors for LR and OS were analyzed on univariable analysis to include age, metastatic tumor size, histologic type, and treatment characteristics. LPFS and overall survival were analyzed using the Kaplan Meier method and log-rank test. Multivariable analysis was performed using Cox proportional hazards model. Multivariable model for LPFS included smoking status, and age. Multivariable model for OS included smoking status, gender, age, tumor size, and chemotherapy. The proportional hazards assumption was tested and was never violated at a p-value of 0.05. Analyses were completed using MedCalc (version 20.1115) and figures were created using Prism (version 9.4.1).

## Results

### Patient characteristics

From 2010 to 2022, 18 patients treated to 26 metastatic lung sarcoma lesions with SBRT were included in this study. Patient characteristics are outlined in Table 1. Of the 18 patients, most were male (72%), had high grade primary sarcoma (66.7%), and had oligometastatic disease at the time of SBRT (72%). Median time from diagnosis to distant metastasis was 21.47 months (range 0.2–175.2). Ten (55.6%) patients had a history of chemotherapy use prior to SBRT. Three (16.7%) patients received prior whole lung irradiation; two patients were treated for Ewing sarcoma and one patient was treated for perihilar primary liposarcoma. Five (27.8%) patients were treated with surgery for initial lung metastases. All patients had stable disease prior to SBRT. The median follow-up time from primary diagnosis was 62.8 months (range 31.3–314.4).

**Table 1:** Patient characteristics (n = 18)

	No	%
Median age at SBRT	61.7	Range 17.1 – 84.8
Sex		
Female	5	27.8
Male	13	72.2
Primary site of disease		
Extremity	6	33.3
Trunk	7	38.9
Pelvis	1	5.6
Head and neck	2	11.1
Retroperitoneum	2	11.1
Primary tumor grade		
Low	0	0
Intermediate	2	11.1
High	12	66.7
Unknown/not graded	4	22.2
Stage at primary diagnosis		
Localized	11	61.1
Metastatic	7	38.9
Smoking status		
Smoker	7	38.9
Never smoked	11	61.1
Median time from primary diagnosis to first distant metastasis, months	21.5	Range 0.2–175.2
Prior sarcoma treatment		
Chemotherapy*	10	55.6
Radiation to lung	3	16.7
Thoracic surgery	5	27.8
Extent disease at time of SBRT treatment		
≤ 5 metastases	13	72.2
> 5 metastases	5	27.8

*Chemotherapy administered for primary disease or at diagnosis of metastasis

### Tumor characteristics and treatment

Characteristics of the lung lesions treated with SBRT are outlined in Table 2. The median number of lesions treated per patient was 1 (range 1–3 lesions). Histology of most lesions were liposarcoma (30.8%), followed by leiomyosarcoma (23.1%) and Ewing/Ewing-like sarcoma (15.4%). Most lesions were in the lower lung lobe (46.2%) and were treated to 50 Gy in 5 fractions (50%). One patient received a dose of 50 Gy in 10 fractions to a hilar lesion at the treating physician’s discretion due to concern for treatment toxicity. The median follow-up time from the end of SBRT was 25.6 months (range 8.2–122.6).

**Table 2:** Lesion characteristics (n = 26)

	No	%
Median number of treated lesions per patient		
Median lesion size, cm	1.1	Range 0.2–3.8
Time from diagnosis to date of SBRT, months		
Histology of primary tumor		
Liposarcoma	8	30.8
Leiomyosarcoma	6	23.1
Ewing/Ewing-like sarcoma	4	15.4
Other*	8	30.8
Location of treated lung lesion		
Lung lobe		
Upper lobe	7	26.9
Lower lobe	12	46.2
Hilar	2	7.7
Middle lobe	5	19.2
Central vs. peripheral		
Central	13	50.0
Peripheral	13	50.0
Ultra-central	0	0
Dose (Gy)/fraction		
54/3	10	38.5
50/5	13	50.0
50/10	1	3.8
42/3	1	3.8
34/1	1	3.8

*Included undifferentiated pleiomorphic sarcoma, myxofibrosarcoma, synovial sarcoma, angiosarcoma, chondrosarcoma

### Local control, local progression-free survival and overall survival

The 1- and 2-year local control rate were 100% and 96%, respectively. Of the 26 metastases, local failure occurred for one lesion (leiomyosarcoma) in a patient at 15.2 months after completion of SBRT to 54 Gy in 3 fractions. LPFS at 2-years was 95.5%. On univariable and multivariable analysis, LPFS was not significantly associated with gender (*p* = 0.48), lesion histology (*p* = 0.42), location in the lung (*p* = 0.37), smoking status (*p* = 0.16), prior chemotherapy (*p* = 0.46), prior radiation to the lung (*p* = 0.055), or oligometastatic versus widespread disease (*p* = 0.6). Multivariable analysis including smoking status, and age did not yield any significant variables.

**Fig. 1:**
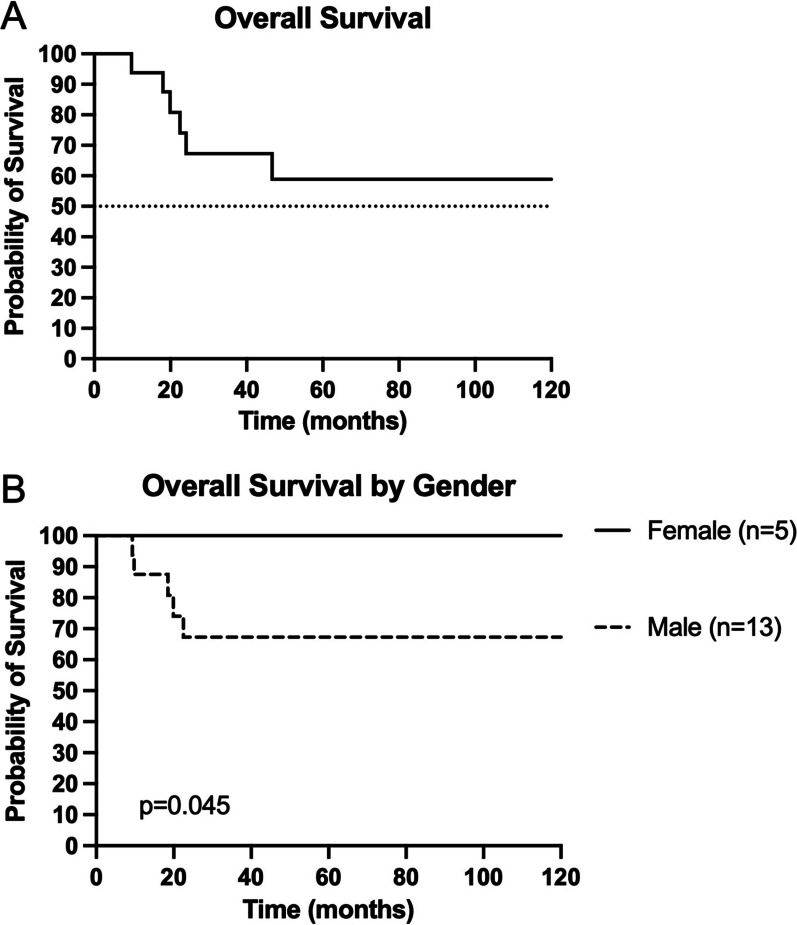
Overall survival

The 2-year OS was 74% and the median OS was not met (Fig. 1a). On univariable analysis, OS significantly differed by gender (*p* = 0.045; Fig. 1b). OS was not associated with primary tumor location (*p* = 0.71), location in the lung (*p* = 0.88), tumor grade (*p* = 0.43), smoking status (*p* = 0.12), patient age at the time of SBRT (*p* = 0.21), prior chemotherapy (*p* = 0.23), prior RT to the lung (*p* = 0.78), or oligometastatic versus widespread disease (*p* = 0.54). Multivariable analysis including smoking status, gender, tumor size, age, and prior chemotherapy did not yield any significant variables.

Of note, analysis excluding the 4 tumors with Ewing/Ewing-like sarcoma, a histology considered to be more radiosensitive, yielded 2-year LPFS and OS of 95% and 71.6%, respectively. Compared to 2-year LPFS and OS of the entire cohort, these rates were not statistically different (*p* = 0.39 and *p* = 0.69, respectively). An additional analysis was performed to exclude the patient treated to 50 Gy 10 fractions and yielded 2-year LPFS and OS of 95.5% and 70.1%, respectively. These rates did not significantly differ from the entire cohort.

### Adverse events

Three patients (16.7%) developed side effects from SBRT and were treated to peripherally (2 patients) and centrally (1 patient) located lesions. All three developed grade 1 fatigue during treatment, however, this did not prevent any from performing their daily activities. One patient who received SBRT to a peripheral lung lesion developed grade 1 nausea without vomiting. There were no adverse events attributed to radiation that were grade 2 or higher.

## Discussion

Sarcoma has long been considered radioresistant, thus surgery is regarded as the primary intervention for localized metastatic disease. Advancing techniques such as SBRT in radiation therapy has enabled broader applications for radiation, which may provide less invasive but still effective local control. In this prospective analysis, we report a 96% local control rate using SBRT for metastatic sarcoma for 26 metastases in 18 patients, with 3 out of 18 patients experiencing grade 1 side effects. Our prospective report adds to the growing body of literature investigating the application of SBRT for local control of metastatic lung sarcoma.

Management for metastatic sarcoma is challenging and often with palliative intent. When deciding treatment modality, consideration of the patient’s symptoms and ability to tolerate treatment side effects are important. Surgery is often the first-line intervention and carries the benefits of excising the tumor, however, it carries high risks for surgical complications such as bleeding, infection, and delayed wound healing. As prognosis for patients with metastatic sarcoma is typically poor with median survival around 33 months after surgical resection [2] providers must take into account the potential for patient recovery and discuss goals of care with the patient. Clinical assessment of progression potential must also be considered and coordinated with systemic therapy use, as surgery requires systemic treatment delay to maximize wound healing. Due to these factors, investigation into SBRT as a treatment option is important, as its non-invasive nature may offer reprieve from surgical complications while still providing adequate local control.

Previous studies have supported the use of SBRT for non-sarcoma pulmonary tumors, including non-small cell lung cancer (NSCLC). Pooled analysis of three randomized control trials comparing SBRT to surgery for metastatic stage 1 NSCLC demonstrated comparable recurrence-free survival rates at 3-years, with 86% in the SBRT group and 80% in the surgery group [11]. These authors also reported fewer grade 3 and 4 adverse events in the SBRT group compared to the surgery group. One retrospective study demonstrated 94% local control rate in 125 NSCLC lesions treated with SBRT at a median follow up time of 19 months. The SABR-COMET Phase 2 randomized trial showed significant overall survival benefit using SBRT for non-sarcomatous oligometastatic metastases, compared to standard of care palliative treatment [12]. Our cohort of patients with sarcoma may serve as an analog to that in the SABR-COMET trial, as they meet the inclusion criteria. Following SBRT for NSCLC, another study reported quality of life remained improved for patients with initially poor baseline, suggesting benefits for SBRT particularly in patients with low performance status. [13]

In contrast to NSCLC, sarcomas are inherently radioresistant, meaning that sarcoma tumor cells have greater capacity for repair after damage from radiation. As such, the effect of SBRT on local control in metastatic sarcoma is less understood and local control rates may be lower than prior data outlining control of non-sarcoma histologies. While it was historically thought that total radiation dose was most impactful for tumoricidal effects, understanding of the importance of radiation treatment schedules involving the fraction dose, dose rate, and overall treatment time have enabled effective tumor targeting [14]. The radiobiology of sarcomas differs from other tumor histologies, as it has a low alpha/beta ratio of around ~ 4–5 (range 0.5–5.5) [7]. The alpha/beta ratio reflects tumor cell sensitivity to the fractionation schedule of radiation; low alpha/beta ratio reflects favorable radiobiologic response and tumor susceptibility to cellular death with high fractionation doses, while greater alpha/beta ratios reflect lower sensitivity of tumor cells to fractionation patterns. Thus, there may be a biologically advantageous effect in escalating the dose per fraction for sarcoma metastases treated with SBRT. This concept provided premise for the present study and, to the authors knowledge, our study is among the first to report prospective data investigating use of SBRT for sarcoma pulmonary tumors. It is worth noting that although sarcomas are collectively viewed as radioresistant, differences in tumor response to radiation exist among sarcoma histologies. Ewing sarcoma, for example, is considered to less radioresistant than other histologies such as leiomyosarcoma and liposarcoma [15]. There were 4 tumors treated with SBRT for metastatic Ewing/Ewing-like sarcoma in the present study. On separate analysis excluding these patients, the LPFS and OS are not statistically different from those including the entire cohort.

Current data exploring the utility of SBRT for metastatic sarcoma are limited to retrospective studies but show promise for its role in the sarcoma treatment paradigm. Our local control rates are consistent with other studies (Additional file 1: Table S1) [6, 16–22]. Navarria et al. [20] reported prospective observational data with 5-year local control rate of 96% in 56 lesions. This group also recently reported a phase II prospective trial in 44 patients with ≤ 4 pulmonary metastasis that were < 5 cm in diameter and not eligible for metastectomy. At a median follow-up of 90 months from diagnosis, the median local recurrence-free survival was 16 months and the 1-year local control was 98.5% [21]. Similarly, Baumann et al. [16] reported a high 2-year local control rate of 90%. Several studies have attempted to retrospectively compare SBRT to surgery for pulmonary metastatic sarcoma. Tetta et al. [23] conducted a systematic review comparing both treatment modalities and reported lower cumulative overall death rate and higher survival rate with disease in the SBRT group than the surgery group. Gutkin et al. [24] reported retrospective data in patients treated for metastatic sarcoma to both intra and extra pulmonary sites, and reported 2-year local control rates of 85% for surgery and 97.7% for SBRT, with 18% and 12% complications for surgery and SBRT, respectively.

Limitations of this study prevent generalizability of the results and include small population size, single institutional nature, and relative short follow up time. Although patients included were prospectively followed, this study did not include age matched controls who underwent other local ablative therapies such as surgical resection, cryoablation, or radiofrequency ablation.

## Conclusion

In this study, stereotactic body radiotherapy for sarcoma pulmonary metastases affords a high rate of local control and low toxicity, consistent with prior sarcoma SBRT retrospective data. This study adds to the wealth of information on SBRT in a radioresistant tumor. Currently, we are aware of 8 studies outlining the role of SBRT for metastatic sarcoma pulmonary metastases, all of which indicate high rates of local control with favorable toxicity profiles. SBRT for pulmonary sarcoma metastases should be carefully considered, especially in the oligometastatic setting, as it offers a non-invasive modality for the management of a tumor historically primarily managed with surgery.

## Supplementary Information

**Additional file 1**: Literature review of studies investigating efficacy of SBRT to pulmonary metastases.

## Data Availability

The datasets used and/or analyzed during the current study are available from the corresponding author on reasonable request.
