# Supplementary material for: Stereotactic body radiotherapy for metastatic sarcoma to the lung: adding to the arsenal of local therapy
Source: Radiat Oncol. 2023 Mar 1;18:42. doi: 10.1186/s13014-023-02226-3 (PMC9976442; doi:10.1186/s13014-023-02226-3)
Supplement: Supplementary file 1 — Additional file 1: Literature review of studies investigating efficacy of SBRT to pulmonary metastases. [file 13014_2023_2226_MOESM1_ESM.docx]

Appendix, Table S1. Literature review of studies investigating efficacy of SBRT to pulmonary metastases

| Author | Year | Type of study | Number of patients and pulmonary lesions | Median Dose/Fraction | Local control (LC) | Overall survival (OS) | Follow up time for LC and OS (years) | Grade 3 Toxicity |
| --- | --- | --- | --- | --- | --- | --- | --- | --- |
| Baumann et al^16^ | 2020 | Retrospective | 44 patients  56 lesions | 24-50/3-5 | 90% | 46% | 2 | 0% |
| Dhakal et al^17^ | 2012 | Retrospective | 14 patients  74 lesions | 55/1-3 | 88% | 50% | 2 | 7% |
| Frakulli et al^18^ | 2015 | Retrospective | 24 patients  68 lesions | 30-60/3-5 | 85.9% | 66.4% | 2 | 0% |
| Lindsay et al^19^ | 2018 | Retrospective | 44 patients  117 lesions | 50/10 | 95% | 82% | 2 | 2.3% |
| Navarria et al^20^ | 2015 | Prospective observational | 28 patients  51 lesions | 48/4 | 96% | 60.5% | 5 | 0% |
| Navarria et al^21^ | 2022 | Prospective | 44 patients  71 lesions | 48-60/3-8 | 98.5% | 66.7% | 1 and 2, respectively | 0% |
| Okunieff et al^6^ | 2006 | Retrospective | 50 patients  125 lesions | 50/5 | 94% | 38% | 2 | 2% |
| Soyfer et al^22^ | 2017 | Retrospective | 22 patients  34 lesions | 60/4 | 100% | 50% | 5 | 2.9% |
| Present study | 2022 | Prospective | 18 patients  26 lesions | 50/5 | 96% | 74% | 2 | 0% |
